# Melatonin attenuates morphine‐induced conditioned place preference in Wistar rats

**DOI:** 10.1002/brb3.2397

**Published:** 2021-10-28

**Authors:** Fahad S. Alshehri, Badrah S. Alghamdi, Alqassem Y. Hakami, Abdullah A. Alshehri, Yusuf S. Althobaiti

**Affiliations:** ^1^ Department of Pharmacology and Toxicology College of Pharmacy Umm Al‐Qura University Makkah Saudi Arabia; ^2^ Department of Physiology Neuroscience Unit Faculty of Medicine King Abdulaziz University Jeddah Saudi Arabia; ^3^ Pre‐Clinical Research Unit King Fahd Medical Research Center King Abdulaziz University Jeddah Saudi Arabia; ^4^ College of Medicine King Saud bin Abdulaziz University for Health Sciences Jeddah Saudi Arabia; ^5^ King Abdullah International Medical Research Center Jeddah Saudi Arabia; ^6^ Department of Pharmacology and Toxicology College of Pharmacy Taif University P.O. Box 11099 Taif 21944 Saudi Arabia; ^7^ College of Pharmacy, Addiction and Neuroscience Research Unit Taif University Taif 21944 Saudi Arabia

**Keywords:** addiction, conditioned place preference, locomotion, melatonin, memory, opioids

## Abstract

**Purpose:**

Morphine is the predominantly used drug for postoperative and cancer pain management. However, the abuse potential of morphine is the primary disadvantage of using opioids in pain management. Melatonin is a neurohormone synthesized in the pineal gland and is involved in circadian rhythms in mammals, as well as other physiological functions. Melatonin provenly attenuates alcohol‐seeking and relapse behaviors in rats. Therefore, we aimed to investigate the involvement of the melatonergic system in attenuating morphine dependence.

**Materials and methods:**

Male Wistar rats were divided into three groups: control, morphine, and morphine + melatonin. Animals were habituated for 3 days, and the initial preference was evaluated. Following the initial preference, the control group received the vehicle and was placed for a 45‐min session in the assigned chamber every day, alternating between the two chambers, for 8 days. The morphine group received a morphine injection (5 mg/kg, IP) and was placed for a 45‐min session in the white chamber, for a total of four sessions. The morphine + melatonin group received the morphine injection (5 mg/kg, IP) for a total of four sessions over an 8‐day period. In the posttest session, the control and morphine groups received a vehicle injection 30 min before placement in the conditioned place preference (CPP). The morphine + melatonin group received a single injection of melatonin (50 mg/kg, IP) 30 min before the preference test.

**Results:**

Statistical analysis revealed that repeated administration of morphine for four sessions produced a significant increase in the CPP score in the morphine group compared to the control group. However, a single melatonin injection administered 30 min before the posttest attenuated morphine‐seeking behavior and reduced morphine‐induced place preference.

**Conclusion:**

These findings provide novel evidence for the role of the melatonergic system as a potential target in modulating morphine‐seeking behavior.

## Graphical abstract

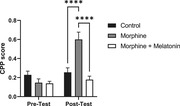

## 1 INTRODUCTION

Morphine is one of the predominantly used drugs in postoperative and cancer pain management (Afsharimani et al., 2011; Aubrun et al., 2003; Donnelly et al., 2002). However, the prolonged use of morphine is associated with an increased probability of tolerance and potential for abuse, which is a primary disadvantage of using opioids in pain management (Norn et al., 2005; Preston et al., 1991). Opioid addiction is a worldwide problem, affecting many countries and causing health concerns due to elevated risks of drug overdose and death (Vadivelu et al., 2018). It has been estimated by the United Nations World Drug Report that around 29.5 million people worldwide are at high‐risk of consumption of opioids drugs (UNODC, 2016). In North America, in countries such as the United States, the opioids public health crisis has led to thousands of deaths (Krausz et al., 2021). In Europe, it has been estimated that around 1.3 million people could be a major risk of opioid addiction in 2018 (Drugs & Addiction, 2018). However, existing treatments for opioid addiction have several limitations, such as poor patient compliance and higher chances of relapse (Corbett et al., 2006; Rosenblum et al., 2008). Exploring alternative drugs to alleviate addiction is thus an urgent necessity for developing targeted and effective treatments (Ballantyne, 2017).

Furthermore, repetitive use of morphine can cause neuroadaptive changes in the brain, leading to seeking behavior upon cessation of use (Spanagel & Shippenberg, 1993; Spanagel et al., 1993). In addition, the nucleus accumbens and ventral tegmental area lie between the main brain regions that undergo neuroadaptive changes due to morphine abuse (Kim et al., 2016). Morphine triggers the opioid G protein‐coupled receptors, which subsequently activate potassium, calcium channels, and adenylate cyclase (Alvarez et al., 2002; Mestek et al., 1995). This is followed by a signaling cascade, including the stimulation of mitogen‐activated protein kinases (MAPK) and extracellular signal‐regulated kinase (ERK) pathways (Dai et al., 2018; Shen et al., 2018).

Cumulative studies have examined the effects of melatonin on opioid tolerance, withdrawal, and hyperalgesia (Cheng et al., 2019; Hemati et al., 2021; Raghavendra & Kulkarni, 1999; Xin et al., 2012). Melatonin is a neurohormone synthesized in the pineal gland and is involved in regulating circadian rhythms in mammals and other physiological functions (Vengeliene et al., 2015). In fact, the co‐administration of melatonin and morphine can delay the development of tolerance to morphine analgesic effects and reverse naloxone‐withdrawal effects (Raghavendra & Kulkarni, 2000). Melatonin receptor antagonists, such as luzindole or prazosin, have failed to reverse morphine tolerance and dependence (Raghavendra & Kulkarni, 2000). Thus, it was suggested that the effect of melatonin on morphine tolerance may be attributed to the inhibitory effect of nitric oxide (Raghavendra & Kulkarni, 2000). Moreover, melatonin provenly attenuates morphine‐induced hyperalgesia by modulating protein kinase C gamma and N‐methyl‐d‐aspartate receptor expression in the spinal cord in rats (Song et al., 2015).

Melatonin activates two G‐coupled protein receptors (MT1 and MT2) that mediate adenylyl cyclase inhibition (von Gall et al., 2002). Melatonin receptors occur in key brain regions, such as the nucleus accumbens, prefrontal cortex, striatum, amygdala, and hippocampus (Musshoff et al., 2002; Uz et al., 2005; Wongprayoon & Govitrapong, 2021). Cumulative studies have established a robust connection between melatonin and seeking behavior associated with drugs of abuse (Conroy et al., 2012; Kovanen et al., 2010; McClung et al., 2005). Moreover, alcohol consumption can disturb circadian rhythms and melatonin production in rats (Peres et al., 2011). Similarly, heroin can affect circadian gene expression, β‐endorphin, and interleukin‐2 (IL‐2) in humans (Li et al., 2009). In addition, methamphetamine use can reduce circadian gene expression in the suprachiasmatic nucleus and striatum in rodents (Iijima et al., 2002; Masubuchi et al., 2000).

Activation of melatonin receptors has shown neuroprotective effects through antioxidant and free radical scavenger properties (Acuña‐Castroviejo et al., 1996; Giusti et al., 1996; D.‐X. Tan, 1993). Furthermore, melatonin attenuates alcohol‐seeking and relapse behaviors in rats (Vengeliene et al., 2015). It has also been reported that melatonin reduces the number of active pokes and cocaine‐seeking behaviors in rats (Takahashi et al., 2017). Thus, the melatonergic system may be a potential target for attenuating drug addiction and dependence. To our knowledge, only a few studies have explored the potential use of melatonin to modulate opioid‐seeking behavior in rats and its role as a potential target in modulating morphine‐seeking behavior using conditioned place preference (CPP).

## 2 MATERIALS AND METHODS

### 2.1 Animals

Male Wistar rats weighing between 280 and 300 g were supplied by King Fahd Medical Research Center, King Abdulaziz University, Jeddah. Two rats were housed in each plastic cage. The temperature was maintained at 21°C, with a room humidity of approximately 50%, and a 12/12 light/dark cycle. All animals had free access to standard food and water. The experiment was performed in accordance with the Animal Care and Use Committee (ACUC) guidelines of King Fahd Medical Research Center. The study experiments were approved by the Biomedical Ethics Research Committee (Reference No. 405−20) at King Abdulaziz University. The experiments adhered to the guidelines of ethics and research on living creatures prepared by the King Abdulaziz City for Science and Technology (KACST), approved by Royal Decree No. M/59 on August 24, 2010.

### 2.2 Drugs

Melatonin (M5250; Sigma Aldrich) (50 mg/kg, intraperitoneal [IP]) (Takahashi et al., 2017) was prepared daily, dissolved in 0.5% ethanol, and diluted with saline. Morphine was supplied by King Abdulaziz University Hospital Pharmacy. The vehicle contained 0.5% ethanol and saline.

### 2.3 Conditioned place preference apparatus

The apparatus comprised two automated Plexiglas designed by Columbus Instruments, Columbus, OH, USA. The two chambers were attached with Auto‐Track software (OPTO‐MAX), which uses infrared (IR) light‐emitting sensors to detect animal movements. The white chamber had vertical white stripes and a smooth white floor. The black chamber had black and white squares and a dotted floor. The time spent, ambulatory count, distance traveled, and resting time were calculated automatically using the OPTO‐MAX software.

### 2.4 Experimental design

**FIGURE 1:**
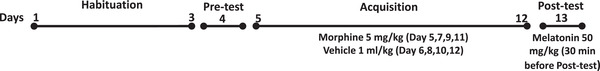
The experimental procedure timeline showing habituation, acquisition, and preference tests

Twenty‐four animals were divided into three groups: control, morphine, and morphine + melatonin. The experiment was performed over 14 days, as shown in Figure 1. All animals were habituated for the first 3 days before commencing the acquisition phase. Habituation was facilitated by placing each animal in the apparatus with both chamber doors opened to allow the animal to freely explore the apparatus for 20 min. All animals showed a preference for the black chamber versus the white chamber in the pretest on Day 4; therefore, we used a biased approach to measure the reward and seeking behavior of morphine.

The acquisition phase was conducted over an 8‐day period (Days 5 to 12) and included four sessions. In the acquisition phase, the control group received the vehicle IP injection and was placed in the assigned chamber for 45 min. The chamber was alternated for each animal. The morphine group received a morphine injection (5 mg/kg, IP) and was placed for 45 min in the white chamber every other day, alternating with the vehicle in the black chamber. The morphine + melatonin group received the morphine injection (5 mg/kg, IP) and was placed for 45 min in the white chamber every other day, alternating with the vehicle in the black chamber. On Day 13 (the posttest day), the control and morphine groups received a vehicle IP injection 30 min before placement in the apparatus. Contrarily, the morphine + melatonin group received melatonin (50 mg/kg, IP) 30 min before testing for preference. Importantly, the animals were placed in the CPP apparatus for the posttest period (20 min).

### 2.5 Statistical analysis

The CPP score was calculated as the time spent in the nonpreferred chamber/total time spent in both chambers, as introduced in a previous study (Sun et al., 2018). The time spent (CPP score), distance traveled, resting time, ambulatory count, and total activity count were analyzed using a two‐way repeated measures analysis of variance (ANOVA), followed by Tukey's post hoc tests. All data were analyzed using Prism 9, and the *p*‐value was set at < .05 for significance.

## 3 RESULTS

### 3.1 Effect of melatonin on morphine‐induced conditioned place preference

**FIGURE 2:**
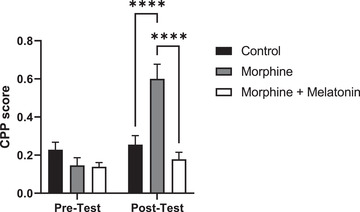
Effect of vehicle, morphine and morphine + melatonin in conditioned place preference (CPP) score on the pretest and posttest. No significant differences were found in the CPP score in pretest between all groups. However, there is a significant increase in the CPP score in the morphine group as compared to the control and morphine + melatonin groups. Moreover, no significant differences were found in CPP score between the control group and the morphine + melatonin group. Values are shown as means ± SEM (****p < .0001) (*n* = 8)

Regarding the CPP score, statistical analyses revealed significant effects of time (*F* [2, 21] = 12.57, *p* = .0003), treatment (*F* [1, 21] = 18.06, *p* = .0004), and treatment × time (*F* [2, 21] = 11.85, *p* = .0004) (Figure 2). Repeated administration of morphine over four sessions produced a significant increase in the CPP score among the morphine group compared to the control group (*p* = .0002). However, a single dose of melatonin administered 30 min before the posttest attenuated morphine‐seeking behavior and prevented morphine‐induced place preference, when comparing the morphine group with the morphine + melatonin group (*p* = < .0001). No significant difference was found between the control and morphine + melatonin groups (*p* = .4866).

**FIGURE 3:**
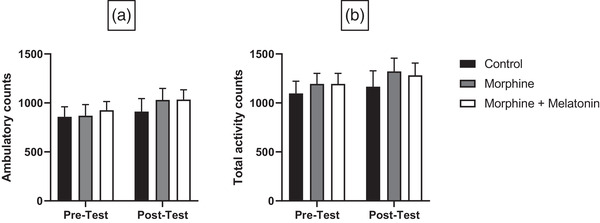
Effect of vehicle, morphine and morphine + melatonin on ambulatory counts (a) and total activity counts (b) in pretest and posttest. Statistical analyses revealed no significant difference in pretest and posttest between all groups in ambulatory counts (a) and total activity counts (b). Values are shown as means ± SEM (*n* = 8)

We then sought to determine other parameters that could potentially affect the interpretation of melatonin on the seeking behavior of morphine using CPP as a tool for seeking and reward measurements. The ambulatory count, total activity, resting time, and distance traveled were measured accordingly. The ambulatory count measured the number of beams broken in the activity plane (the CPP apparatus). When beams were broken, the program saved this information as counts, except for stereotypic movements associated with scratching or grooming, which were not counted. Regarding the ambulatory count, no significant effects were observed for treatment (*F* [1, 21] = 2.323, *p* = .1424), time (*F* [2, 21] = 0.2787, *p* = .7596), or treatment × time (*F* [2, 21] = 0.1920, *p* = .8267) (Figure 3a). The total activity tallied every broken beam within the activity plane, including stereotypic movement. Analysis of the total activity revealed no significant effects of treatment (*F* [1, 21] = 0.9263, *p* = .3468), time (*F* [2, 21] = 0.5048, *p* = .6107), or treatment × time (*F* [2, 21] = 0.03112, *p* = .9694) (Figure 3b).

**FIGURE 4:**
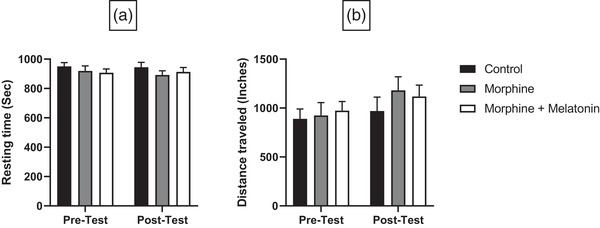
Effect of vehicle, morphine and morphine + melatonin on resting time (a) and distance travelled (b) in pretest and posttest. Statistical analyses exhibited no significant difference in pretest and posttest between all groups in resting time (a) and distance travelled (b). Values are shown as means ± SEM (*n* = 8)

The resting time represented periods when the animals were not moving. Statistical analyses showed no significant effects of treatment (*F* [1, 21]= 0.2192, *p* = .6445), time (*F* [2, 21] = 0.8675, *p* = 0.4345), or treatment × time (*F* [2, 21] = 0.2793, *p* = .7591) (Figure 4a). The distance traveled was calculated in inches for the entire testing period. Statistical analyses indicated a significant effect of treatment (*F* {1, 21] = 4.393, *p* = 0.0484), but no significant effect for time (*F* [2, 21] = 0.4612, *p* = .6368) and treatment × time (*F* [2, 21] = 0.4459, *p* = .6462) (Figure 4b).

## 4 DISCUSSION

This study aimed to investigate the involvement of the melatonergic system in attenuating morphine dependence. Over time, several methods and tools have been developed to measure motivational drug rewards in animals, drawing on the concept of Pavlovian conditioning in animals, including CPP and self‐administration techniques (Achat‐Mendes et al., 2005; Beckmann et al., 2011; Bryant et al., 2009; Krank, 2003). We employed the CPP tool because it is less stressful for animals and does not require the surgical implantation of a catheter, such as in the self‐administration paradigm and the extensive operant training history (Prus et al., 2009). In addition, opioids have generally produced consistent reward effects using the CPP paradigm (Alshehri et al., 2018; Ashby et al., 2003; Mueller et al., 2002; Niikura et al., 2013). This study used CPP to measure the effect of melatonin on morphine‐induced place preference. Moreover, the CPP paradigm provided greater insight into animal behavior during the posttest, such as measuring the ambulatory count, total activity, resting time, and total distance traveled. Interestingly, repeated administration of morphine over four sessions produced a significant increase in the CPP score. However, melatonin attenuated morphine‐seeking behavior and prevented morphine‐induced place preference. Therefore, melatonin may be a potential drug to modulate morphine‐associated seeking effects and dependence.

Opioid addiction is a global issue with devastating social and health consequences (Volkow et al., 2019). Exploring potential alternatives for the management of opioid abuse is thus a critical necessity. Melatonin exerts its physiological effects by activating melatonin receptors 1 and 2 (Onaolapo & Onaolapo, 2018). Researchers have focused on exploring the potential roles of melatonin in addiction, including dopaminergic system involvement, such as seeking behaviors or dependence (Vengeliene et al., 2015). Several studies indicated that melatonin could reduce dopamine release as an inhibitory effect through melatonin receptors (Zisapel, 2001; Zisapel et al., 1982, 1983). Other studies have shown that repeated melatonin administration can provide neuroprotective effects against dopamine‐induced degeneration through pro‐inflammatory cytokines and upregulate antioxidant enzyme expression in homozygous zitter (zi/zi) rats (Hashimoto et al., 2012). Dopamine neurotransmission in the nucleus accumbens plays a critical role in opioid dependence (Di Chiara et al., 1999; Willuhn et al., 2010). The interactions between melatonin and the dopaminergic systems suggest that melatonin could be a potential modulator of opioid‐seeking behavior (Motaghinejad et al., 2015; Uz et al., 2005; Yahyavi‐Firouz‐Abadi et al., 2007). Thus, this study demonstrated that melatonin could attenuate morphine‐seeking behavior, in part, through its interaction with dopamine neurotransmission.

Cumulative studies have explored the effects of opioids on the induction of inflammation and antioxidant enzyme activity (Xu et al., 2006; Zhang et al., 2004; Zhou et al., 2001). Findings indicate that morphine increases the activity of oxidative damage molecules, such as 8‐hydroxydeoxyguanosine and other related biomolecules in animals (Zhang et al., 2004). In addition, opioids provenly reduce the activity of in vivo antioxidative enzymes, such as glutathione, and activities of superoxide dismutase, glutathione peroxidase, and catalase antioxidant enzymes (Payabvash et al., 2006; Singhal et al., 1994; Zhang et al., 2004). Contrarily, studies have suggested that melatonin may enhance the antioxidant effect by increasing mRNA expression (Reiter et al., 2003; Reiter et al., 2000). Melatonin has also been shown to enhance many antioxidative enzymes, such as glutathione reductase enzyme, glutathione peroxidase, and superoxide dismutase (Tomas‐Zapico & Coto‐Montes, 2005). In addition, melatonin has free radical scavenger properties on hydroxyl radicals and superoxide ion radicals in in vivo and in vitro models (D. Tan et al., 2002). Thus, melatonin can exert a regulatory effect on antioxidant enzymes and may play a role in modulating morphine‐seeking behavior.

Furthermore, melatonin has been used as a hypnotic, resynchronizing, and antioxidant agent in clinical practice (Maldonado et al., 2009). Melatonin is associated with regulation of the circadian sleep‐wake rhythm and circadian secretion of hormones (Pandi‐Perumal et al., 2008). Several studies have suggested that exogenously administered melatonin has sedative‐hypnotic properties in humans (Naguib et al., 2003; Shavali et al., 2005). Moreover, melatonin may produce anxiolytic and sedation effects in adults and children, without affecting motor ability or impacting recovery (Naguib & Samarkandi, 1999, 2000). Conversely, while cumulative studies have reported that exogenously administered melatonin can produce a sedation effect in animals, other studies have reported no sleep‐related effects (Wang et al., 2003, 2002). Some studies reported that melatonin did not induce a sedation effect (Dyche et al., 2012; Fisher & Sugden, 2010), whereas others found that in low doses, melatonin could enhance sedation in rats (Mendelson, 2002; Mendelson et al., 1980). Thus, this study measured ambulatory count, total activity, resting time, and distance traveled to address the sedation hypothesis. These parameters provided a detailed understanding of animal behavior following melatonin injection and during the posttest period. Statistical analyses revealed that melatonin administration 30 min before the posttest did not affect these parameters, compared to the morphine and morphine + melatonin groups. Therefore, the melatonin attenuation of morphine‐seeking behavior was not attributable to the sedative‐hypnotic effects of melatonin.

## 5 CONCLUSION

This study showed the effect of melatonin on morphine seeking behavior using the CPP paradigm. Morphine produces a seeking effect using the CPP paradigm. Interestingly, melatonin attenuated the morphine‐induced preference when injected 30 minutes before the posttest. The melatonin effect may be attributed to its interaction with dopamine neurotransmission and its modulatory effect on antioxidant enzymes in the brain. However, we provided evidence that the melatonin effect was distinct from its sedative‐hypnotic properties. Further studies are warranted to investigate the molecular effect of melatonin on dopamine neurotransmission in the nucleus accumbens and other brain regions.

## CONFLICT OF INTEREST

The authors declare no conflict of interest.

## FUNDING INFORMATION

Deanship of Scientific Research, Grant Number: G: 621‐140‐1441.

### TRANSPARENT PEER REVIEW

The peer review history for this article is available at https://publons.com/publon/10.1002/brb3.2397

## Data Availability

The datasets generated during and analyzed during the current study are available from the corresponding author on reasonable request

## References

[brb32397-bib-0001] Achat‐Mendes, C. , Ali, S. F. , & Itzhak, Y. (2005). Differential effects of amphetamines‐induced neurotoxicity on appetitive and aversive Pavlovian conditioning in mice. Neuropsychopharmacology, 30(6), 1128–1137. 10.1038/sj.npp.1300675 15688084

[brb32397-bib-0002] Acuña‐Castroviejo, D. , Coto‐Montes, A. , Monti, M. G. , Ortiz, G. G. , & Reiter, R. J. (1996). Melatonin is protective against MPTP‐induced striatal and hippocampal lesions. Life Sciences, 60(2), PL23–PL29. 10.1016/S0024-3205(96)00606-6 9000122

[brb32397-bib-0003] Afsharimani, B. , Cabot, P. J. , & Parat, M. O. (2011). Morphine use in cancer surgery. Frontiers in Pharmacology, 2, 46. 10.3389/fphar.2011.00046 21852973 PMC3151591

[brb32397-bib-0004] Alshehri, F. S. , Hakami, A. Y. , Althobaiti, Y. S. , & Sari, Y. (2018). Effects of ceftriaxone on hydrocodone seeking behavior and glial glutamate transporters in P rats. Behavioural Brain Research, 347, 368–376. 10.1016/j.bbr.2018.03.043 29604365 PMC5988953

[brb32397-bib-0005] Alvarez, V. A. , Arttamangkul, S. , Dang, V. , Salem, A. , Whistler, J. L. , Von Zastrow, M. , Grandy, D. K. , & Williams, J. T. (2002). mu‐Opioid receptors: Ligand‐dependent activation of potassium conductance, desensitization, and internalization. Journal of Neuroscience, 22(13), 5769–5776. 10.1523/JNEUROSCI.22-13-05769.2002 12097530 PMC6758217

[brb32397-bib-0006] Ashby, C. R. Jr. , Paul, M. , Gardner, E. L. , Heidbreder, C. A. , & Hagan, J. J. (2003). Acute administration of the selective D3 receptor antagonist SB‐277011A blocks the acquisition and expression of the conditioned place preference response to heroin in male rats. Synapse, 48(3), 154–156. 10.1002/syn.10188 12645041

[brb32397-bib-0007] Aubrun, F. , Bunge, D. , Langeron, O. , Saillant, G. , Coriat, P. , & Riou, B. (2003). Postoperative morphine consumption in the elderly patient. Anesthesiology, 99(1), 160–165. 10.1097/00000542-200307000-00026 12826856

[brb32397-bib-0008] Ballantyne, J. C. (2017). Opioids for the treatment of chronic pain: Mistakes made, lessons learned, and future directions. Anesthesia and Analgesia, 125(5), 1769–1778. 10.1213/ANE.0000000000002500 29049121

[brb32397-bib-0009] Beckmann, J. S. , Marusich, J. A. , Gipson, C. D. , & Bardo, M. T. (2011). Novelty seeking, incentive salience and acquisition of cocaine self‐administration in the rat. Behavioural Brain Research, 216(1), 159–165. 10.1016/j.bbr.2010.07.022 20655954 PMC2975769

[brb32397-bib-0010] Bryant, C. D. , Roberts, K. W. , Culbertson, C. S. , Le, A. , Evans, C. J. , & Fanselow, M. S. (2009). Pavlovian conditioning of multiple opioid‐like responses in mice. Drug and Alcohol Dependence, 103(1–2), 74–83. 10.1016/j.drugalcdep.2009.03.016 19419821 PMC3085957

[brb32397-bib-0011] Cheng, Y. C. , Tsai, R. Y. , Sung, Y. T. , Chen, I. J. , Tu, T. Y. , Mao, Y. Y. , & Wong, C. S. (2019). Melatonin regulation of transcription in the reversal of morphine tolerance: Microarray analysis of differential gene expression. International Journal of Molecular Medicine, 43(2), 791–806. 10.3892/ijmm.2018.4030 30569162 PMC6317689

[brb32397-bib-0012] Conroy, D. A. , Hairston, I. S. , Arnedt, J. T. , Hoffmann, R. F. , Armitage, R. , & Brower, K. J. (2012). Dim light melatonin onset in alcohol‐dependent men and women compared with healthy controls. Chronobiology International, 29(1), 35–42. 10.3109/07420528.2011.636852 22217099 PMC4258345

[brb32397-bib-0013] Corbett, A. D. , Henderson, G. , McKnight, A. T. , & Paterson, S. J. (2006). 75 years of opioid research: The exciting but vain quest for the Holy Grail. British Journal of Pharmacology, 147(Suppl 1), S153–S162. 10.1038/sj.bjp.0706435 PMC1760732 16402099

[brb32397-bib-0014] Dai, W. L. , Liu, X. T. , Bao, Y. N. , Yan, B. , Jiang, N. , Yu, B. Y. , & Liu, J. H. (2018). Selective blockade of spinal D2DR by levo‐corydalmine attenuates morphine tolerance via suppressing PI3K/Akt‐MAPK signaling in a MOR‐dependent manner. Experimental & Molecular Medicine, 50(11), 1–12. 10.1038/s12276-018-0175-1 PMC6235923 30429454

[brb32397-bib-0015] Di Chiara, G. , Tanda, G. , Bassareo, V. , Pontieri, F. , Acquas, E. , Fenu, S. , Cadoni, C. , & Carboni, E. (1999). Drug addiction as a disorder of associative learning: Role of nucleus accumbens shell/extended amygdala dopamine. Annals of the New York Academy of Sciences, 877(1), 461–485. 10.1111/j.1749-6632.1999.tb09283.x 10415665

[brb32397-bib-0016] Donnelly, S. , Davis, M. P. , Walsh, D. , Naughton, M. , & World Health, O. (2002). Morphine in cancer pain management: A practical guide. Supportive Care in Cancer, 10(1), 13–35. 10.1007/s005200100274 11777184

[brb32397-bib-0017] Drugs, E. M. C. F. , & Addiction, D. (2018). European drug report 2018: Trends and developments. Office for Official Publications of the European Communities.

[brb32397-bib-0018] Dyche, J. , Anch, A. M. , Fogler, K. A. , Barnett, D. W. , & Thomas, C. (2012). Effects of power frequency electromagnetic fields on melatonin and sleep in the rat. Emerging Health Threats Journal, 5(1), 10904. 10.3402/ehtj.v5i0.10904 PMC3334267 22529876

[brb32397-bib-0019] Fisher, S. P. , & Sugden, D. (2010). Endogenous melatonin is not obligatory for the regulation of the rat sleep‐wake cycle. Sleep, 33(6), 833–840. 10.1093/sleep/33.6.833 20550025 PMC2881717

[brb32397-bib-0020] Giusti, P. , Lipartiti, M. , Franceschini, D. , Schiavo, N. , Floreani, M. , & Manev, H. (1996). Neuroprotection by melatonin from kainate‐induced excitotoxicity in rats. FASEB Journal, 10(8), 891–896. 10.1096/fasebj.10.8.8666166 8666166

[brb32397-bib-0021] Hashimoto, K. , Ueda, S. , Ehara, A. , Sakakibara, S. , Yoshimoto, K. , & Hirata, K. (2012). Neuroprotective effects of melatonin on the nigrostriatal dopamine system in the zitter rat. Neuroscience Letters, 506(1), 79–83. 10.1016/j.neulet.2011.10.053 22056485

[brb32397-bib-0022] Hemati, K. , Pourhanifeh, M. H. , Dehdashtian, E. , Fatemi, I. , Mehrzadi, S. , Reiter, R. J. , & Hosseinzadeh, A. (2021). Melatonin and morphine: Potential beneficial effects of co‐use. Fundamental and Clinical Pharmacology, 35(1), 25–39. 10.1111/fcp.12566 32415694

[brb32397-bib-0023] Iijima, M. , Nikaido, T. , Akiyama, M. , Moriya, T. , & Shibata, S. (2002). Methamphetamine‐induced, suprachiasmatic nucleus‐independent circadian rhythms of activity and mPer gene expression in the striatum of the mouse. European Journal of Neuroscience, 16(5), 921–929. 10.1046/j.1460-9568.2002.02140.x 12372028

[brb32397-bib-0024] Kim, J. , Ham, S. , Hong, H. , Moon, C. , & Im, H. I. (2016). Brain reward circuits in morphine addiction. Molecules and Cells, 39(9), 645–653. 10.14348/molcells.2016.0137 27506251 PMC5050528

[brb32397-bib-0025] Kovanen, L. , Saarikoski, S. T. , Haukka, J. , Pirkola, S. , Aromaa, A. , Lonnqvist, J. , & Partonen, T. (2010). Circadian clock gene polymorphisms in alcohol use disorders and alcohol consumption. Alcohol and Alcoholism, 45(4), 303–311. 10.1093/alcalc/agq035 20554694

[brb32397-bib-0026] Krank, M. D. (2003). Pavlovian conditioning with ethanol: Sign‐tracking (autoshaping), conditioned incentive, and ethanol self‐administration. Alcoholism, Clinical and Experimental Research, 27(10), 1592–1598. 10.1097/01.ALC.0000092060.09228.DE 14574229

[brb32397-bib-0027] Krausz, R. M. , Westenberg, J. N. , & Ziafat, K. (2021). The opioid overdose crisis as a global health challenge. Current Opinion in Psychiatry, 34(4), 405–412. 10.1097/YCO.0000000000000712 33901060

[brb32397-bib-0028] Li, S. X. , Shi, J. , Epstein, D. H. , Wang, X. , Zhang, X. L. , Bao, Y. P. , Zhang, D. , Zhang, X.‐Y. , Kosten, T. R. , & Lu, L. (2009). Circadian alteration in neurobiology during 30 days of abstinence in heroin users. Biological Psychiatry, 65(10), 905–912. 10.1016/j.biopsych.2008.11.025 19135652

[brb32397-bib-0029] Maldonado, M. D. , Perez‐San‐Gregorio, M. A. , & Reiter, R. J. (2009). The role of melatonin in the immuno‐neuro‐psychology of mental disorders. Recent Patents on CNS Drug Discovery, 4(1), 61–69. 10.2174/157488909787002564 19149715

[brb32397-bib-0030] Masubuchi, S. , Honma, S. , Abe, H. , Ishizaki, K. , Namihira, M. , Ikeda, M. , & Honma, K. (2000). Clock genes outside the suprachiasmatic nucleus involved in manifestation of locomotor activity rhythm in rats. European Journal of Neuroscience, 12(12), 4206–4214. https://www.ncbi.nlm.nih.gov/pubmed/11122332 11122332

[brb32397-bib-0031] McClung, C. A. , Sidiropoulou, K. , Vitaterna, M. , Takahashi, J. S. , White, F. J. , Cooper, D. C. , & Nestler, E. J. (2005). Regulation of dopaminergic transmission and cocaine reward by the Clock gene. Proceedings of the National Academy of Sciences of the United States of America, 102(26), 9377–9381. 10.1073/pnas.0503584102 15967985 PMC1166621

[brb32397-bib-0032] Mendelson, W. B. (2002). Melatonin microinjection into the medial preoptic area increases sleep in the rat. Life Sciences, 71, 2067–2070. 10.1016/S0024-3205(02)01991-4 12175899

[brb32397-bib-0033] Mendelson, W. B. , Gillin, J. C. , Dawson, S. D. , Lewy, A. J. , & Wyatt, R. J. (1980). Effects of melatonin and propranolol on sleep of the rat. Brain Research, 201(1), 240–244. 10.1016/0006-8993(80)90793-3 7417837

[brb32397-bib-0034] Mestek, A. , Hurley, J. H. , Bye, L. S. , Campbell, A. D. , Chen, Y. , Tian, M. , Liu, J. , Schulman, H. , & Yu, L. (1995). The human mu opioid receptor: Modulation of functional desensitization by calcium/calmodulin‐dependent protein kinase and protein kinase C. Journal of Neuroscience, 15(3 Pt 2), 2396–2406. https://www.ncbi.nlm.nih.gov/pubmed/7891175, 10.1523/JNEUROSCI.15-03-02396.1995 7891175 PMC6578163

[brb32397-bib-0035] Motaghinejad, M. , Motaghinejad, O. , & Hosseini, P. (2015). Attenuation of morphine physical dependence and blood levels of cortisol by central and systemic administration of ramelteon in rat. Iranian Journal of Medical Sciences, 40(3), 240–247. https://www.ncbi.nlm.nih.gov/pubmed/25999624 25999624 PMC4430886

[brb32397-bib-0036] Mueller, D. , Perdikaris, D. , & Stewart, J. (2002). Persistence and drug‐induced reinstatement of a morphine‐induced conditioned place preference. Behavioural Brain Research, 136(2), 389–397. 10.1016/S0166-4328(02)00297-8 12429400

[brb32397-bib-0037] Musshoff, U. , Riewenherm, D. , Berger, E. , Fauteck, J. D. , & Speckmann, E. J. (2002). Melatonin receptors in rat hippocampus: Molecular and functional investigations. Hippocampus, 12(2), 165–173. 10.1002/hipo.1105 12000116

[brb32397-bib-0038] Naguib, M. , Hammond, D. L. , Schmid, P. G. 3rd , Baker, M. T. , Cutkomp, J. , Queral, L. , & Smith, T. (2003). Pharmacological effects of intravenous melatonin: Comparative studies with thiopental and propofol. British Journal of Anaesthesia, 90(4), 504–507. 10.1093/bja/aeg092 12644425

[brb32397-bib-0039] Naguib, M. , & Samarkandi, A. H. (1999). Premedication with melatonin: A double‐blind, placebo‐controlled comparison with midazolam. British Journal of Anaesthesia, 82(6), 875–880. 10.1093/bja/82.6.875 10562782

[brb32397-bib-0040] Naguib, M. , & Samarkandi, A. H. (2000). The comparative dose‐response effects of melatonin and midazolam for premedication of adult patients: A double‐blinded, placebo‐controlled study. Anesthesia and Analgesia, 91(2), 473–479. 10.1097/00000539-200008000-00046 10910871

[brb32397-bib-0041] Niikura, K. , Ho, A. , Kreek, M. J. , & Zhang, Y. (2013). Oxycodone‐induced conditioned place preference and sensitization of locomotor activity in adolescent and adult mice. Pharmacology, Biochemistry and Behavior, 110, 112–116. 10.1016/j.pbb.2013.06.010 PMC3805792 23827650

[brb32397-bib-0042] Norn, S. , Kruse, P. R. , & Kruse, E. (2005). History of opium poppy and morphine. Dan Medicinhist Arbog, 33, 171–184. https://www.ncbi.nlm.nih.gov/pubmed/17152761 17152761

[brb32397-bib-0043] Onaolapo, O. , & Onaolapo, A. (2018). Melatonin: Medical uses and role in health and disease. *Melatonin receptors, behaviour and brain function* (pp. 133–158). Nova Science Publishers.

[brb32397-bib-0044] Pandi‐Perumal, S. R. , Trakht, I. , Spence, D. W. , Srinivasan, V. , Dagan, Y. , & Cardinali, D. P. (2008). The roles of melatonin and light in the pathophysiology and treatment of circadian rhythm sleep disorders. Nature Clinical Practice Neurology, 4(8), 436–447. 10.1038/ncpneuro0847 18628753

[brb32397-bib-0045] Payabvash, S. , Beheshtian, A. , Salmasi, A. H. , Kiumehr, S. , Ghahremani, M. H. , Tavangar, S. M. , Sabzevari, O. , & Dehpour, A. R. (2006). Chronic morphine treatment induces oxidant and apoptotic damage in the mice liver. Life Sciences, 79(10), 972–980. 10.1016/j.lfs.2006.05.008 16750225

[brb32397-bib-0046] Peres, R. , do Amaral, F. G. , Madrigrano, T. C. , Scialfa, J. H. , Bordin, S. , Afeche, S. C. , & Cipolla‐Neto, J. (2011). Ethanol consumption and pineal melatonin daily profile in rats. Addiction Biology, 16(4), 580–590. 10.1111/j.1369-1600.2011.00342.x 21635669

[brb32397-bib-0047] Preston, K. L. , Jasinski, D. R. , & Testa, M. (1991). Abuse potential and pharmacological comparison of tramadol and morphine. Drug and Alcohol Dependence, 27(1), 7—17. 10.1016/0376-8716(91)90081-9 2029860

[brb32397-bib-0048] Prus, A. J. , James, J. R. , & Rosecrans, J. A. (2009). Conditioned place preference. In J. J. Buccafusco (Ed.), Methods of behavioral analysis in neuroscience (pp. 59–76). CRC Press/Routledge/Taylor & Francis Group. 21204336

[brb32397-bib-0049] Raghavendra, V. , & Kulkarni, S. K. (1999). Reversal of morphine tolerance and dependence by melatonin: Possible role of central and peripheral benzodiazepine receptors. Brain Research, 834(1–2), 178–181. 10.1016/S0006-8993(99)01520-6 10407111

[brb32397-bib-0050] Raghavendra, V. , & Kulkarni, S. K. (2000). Possible mechanisms of action in melatonin reversal of morphine tolerance and dependence in mice. European Journal of Pharmacology, 409(3), 279–289. 10.1016/S0014-2999(00)00849-9 11108822

[brb32397-bib-0051] Reiter, R. J. , Tan, D. X. , Mayo, J. C. , Sainz, R. M. , Leon, J. , & Czarnocki, Z. (2003). Melatonin as an antioxidant: Biochemical mechanisms and pathophysiological implications in humans. Acta Biochimica Polonica, 50(4), 1129–1146. 10.18388/abp.2003_3637 14740000

[brb32397-bib-0052] Reiter, R. J. , Tan, D. X. , Osuna, C. , & Gitto, E. (2000). Actions of melatonin in the reduction of oxidative stress. A review. Journal of Biomedical Science, 7(6), 444–458. 10.1007/BF02253360 11060493

[brb32397-bib-0053] Rosenblum, A. , Marsch, L. A. , Joseph, H. , & Portenoy, R. K. (2008). Opioids and the treatment of chronic pain: Controversies, current status, and future directions. Experimental and Clinical Psychopharmacology, 16(5), 405–416. 10.1037/a0013628 18837637 PMC2711509

[brb32397-bib-0054] Shavali, S. , Ho, B. , Govitrapong, P. , Sawlom, S. , Ajjimaporn, A. , Klongpanichapak, S. , & Ebadi, M. (2005). Melatonin exerts its analgesic actions not by binding to opioid receptor subtypes but by increasing the release of beta‐endorphin an endogenous opioid. Brain Research Bulletin, 64(6), 471–479. 10.1016/j.brainresbull.2004.09.008 15639542

[brb32397-bib-0055] Shen, L. , Wang, W. , Li, S. , Qin, J. , & Huang, Y. (2018). NMDA receptor antagonists attenuate intrathecal morphine‐induced pruritus through ERK phosphorylation. Molecular Brain, 11(1), 35. 10.1186/s13041-018-0379-2 29954440 PMC6022508

[brb32397-bib-0056] Singhal, P. C. , Pamarthi, M. , Shah, R. , Chandra, D. , & Gibbons, N. (1994). Morphine stimulates superoxide formation by glomerular mesangial cells. Inflammation, 18(3), 293–299 10.1007/BF01534270 8088925

[brb32397-bib-0057] Song, L. , Wu, C. , & Zuo, Y. (2015). Melatonin prevents morphine‐induced hyperalgesia and tolerance in rats: Role of protein kinase C and N‐methyl‐D‐aspartate receptors. BMC Anesthesiology, 15(1), 12. 10.1186/1471-2253-15-12 25745356 PMC4350305

[brb32397-bib-0058] Spanagel, R. , Almeida, O. F. , & Shippenberg, T. S. (1993). Long lasting changes in morphine‐induced mesolimbic dopamine release after chronic morphine exposure. Synapse, 14(3), 243–245. 10.1002/syn.890140307 8211708

[brb32397-bib-0059] Spanagel, R. , & Shippenberg, T. S. (1993). Modulation of morphine‐induced sensitization by endogenous kappa opioid systems in the rat. Neuroscience Letters, 153(2), 232–236. 10.1016/0304-3940(93)90329-J 8392157

[brb32397-bib-0060] Sun, Y. , Chen, G. , Zhou, K. , & Zhu, Y. (2018). A conditioned place preference protocol for measuring incubation of craving in rats. Journal of Visualized Experiments, 141, e58384. 10.3791/58384 30474645

[brb32397-bib-0061] Takahashi, T. T. , Vengeliene, V. , & Spanagel, R. (2017). Melatonin reduces motivation for cocaine self‐administration and prevents relapse‐like behavior in rats. Psychopharmacology, 234(11), 1741–1748. 10.1007/s00213-017-4576-y 28246896

[brb32397-bib-0062] Tan, D.‐X. (1993). Melatonin: A potent, endogenous hydroxyl radical scavenger. Endocrine Journal, 1, 57–60.

[brb32397-bib-0063] Tan, D. , Reiter, R. J. , Manchester, L. C. , Yan, M. , El‐Sawi, M. , Sainz, R. M. , Mayo, J. C. , Kohen, R. , Allegra, M. , & Hardeland, R. (2002). Chemical and physical properties and potential mechanisms: Melatonin as a broad spectrum antioxidant and free radical scavenger. Current Topics in Medicinal Chemistry, 2(2), 181–197. 10.2174/1568026023394443 11899100

[brb32397-bib-0064] Tomas‐Zapico, C. , & Coto‐Montes, A. (2005). A proposed mechanism to explain the stimulatory effect of melatonin on antioxidative enzymes. Journal of Pineal Research, 39(2), 99–104. 10.1111/j.1600-079X.2005.00248.x 16098085

[brb32397-bib-0065] UNODC, U. (2016). World drug report 2013. United Nations publication.

[brb32397-bib-0066] Uz, T. , Arslan, A. D. , Kurtuncu, M. , Imbesi, M. , Akhisaroglu, M. , Dwivedi, Y. , Pandey, G. N. , & Manev, H. (2005). The regional and cellular expression profile of the melatonin receptor MT1 in the central dopaminergic system. Brain Research Molecular Brain Research, 136(1–2), 45–53. 10.1016/j.molbrainres.2005.01.002 15893586

[brb32397-bib-0067] Vadivelu, N. , Kai, A. M. , Kodumudi, V. , Sramcik, J. , & Kaye, A. D. (2018). The opioid crisis: A comprehensive overview. Current Pain and Headache Reports, 22(3), 16. 10.1007/s11916-018-0670-z 29476358

[brb32397-bib-0068] Vengeliene, V. , Noori, H. R. , & Spanagel, R. (2015). Activation of melatonin receptors reduces relapse‐like alcohol consumption. Neuropsychopharmacology, 40(13), 2897–2906. 10.1038/npp.2015.143 25994077 PMC4864625

[brb32397-bib-0069] Volkow, N. D. , Icaza, M. E. M. , Poznyak, V. , Saxena, S. , Gerra, G. , & Network, U.‐W. I. S. (2019). Addressing the opioid crisis globally. World Psychiatry, 18(2), 231–232. 10.1002/wps.20633 31059614 PMC6502427

[brb32397-bib-0070] von Gall, C. , Stehle, J. H. , & Weaver, D. R. (2002). Mammalian melatonin receptors: Molecular biology and signal transduction. Cell and Tissue Research, 309(1), 151–162. 10.1007/s00441-002-0581-4 12111545

[brb32397-bib-0071] Wang, F. , Li, J. , Wu, C. , Yang, J. , Xu, F. , & Zhao, Q. (2003). The GABAA receptor mediates the hypnotic activity of melatonin in rats. Pharmacology Biochemistry and Behavior, 74(3), 573–578. 10.1016/S0091-3057(02)01045-6 12543221

[brb32397-bib-0072] Wang, F. , Li, J. C. , Wu, C. F. , Yang, J. Y. , Xu, F. , & Peng, F. (2002). Hypnotic activity of melatonin: Involvement of semicarbazide hydrochloride, blocker of synthetic enzyme for GABA. Acta Pharmacologica Sinica, 23(9), 860–864. https://www.ncbi.nlm.nih.gov/pubmed/12230959 12230959

[brb32397-bib-0073] Willuhn, I. , Wanat, M. J. , Clark, J. J. , & Phillips, P. E. (2010). Dopamine signaling in the nucleus accumbens of animals self‐administering drugs of abuse. Current Topics in Behavioral Neurosciences, 3, 29–71. 10.1007/7854_2009_27 21161749 PMC3766749

[brb32397-bib-0074] Wongprayoon, P. , & Govitrapong, P. (2021). Melatonin receptor as a drug target for neuroprotection. Current Molecular Pharmacology, 14(2), 150–164. 10.2174/1874467213666200421160835 32316905

[brb32397-bib-0075] Xin, W. , Chun, W. , Ling, L. , & Wei, W. (2012). Role of melatonin in the prevention of morphine‐induced hyperalgesia and spinal glial activation in rats: Protein kinase C pathway involved. International Journal of Neuroscience, 122(3), 154–163. 10.3109/00207454.2011.635828 22050217

[brb32397-bib-0076] Xu, B. , Wang, Z. , Li, G. , Li, B. , Lin, H. , Zheng, R. , & Zheng, Q. (2006). Heroin‐administered mice involved in oxidative stress and exogenous antioxidant‐alleviated withdrawal syndrome. Basic & Clinical Pharmacology & Toxicology, 99(2), 153–161. 10.1111/j.1742-7843.2006.pto_461.x 16918717

[brb32397-bib-0077] Yahyavi‐Firouz‐Abadi, N. , Tahsili‐Fahadan, P. , Ghahremani, M. H. , & Dehpour, A. R. (2007). Melatonin enhances the rewarding properties of morphine: Involvement of the nitric oxidergic pathway. Journal of Pineal Research, 42(4), 323–329. 10.1111/j.1600-079X.2007.00422.x 17439548

[brb32397-bib-0078] Zhang, Y. T. , Zheng, Q. S. , Pan, J. , & Zheng, R. L. (2004). Oxidative damage of biomolecules in mouse liver induced by morphine and protected by antioxidants. Basic & Clinical Pharmacology & Toxicology, 95(2), 53–58. 10.1111/j.1742-7843.2004.950202.x 15379780

[brb32397-bib-0079] Zhou, J. , Si, P. , Ruan, Z. , Ma, S. , Yan, X. , Sun, L. , Peng, F. , Yuan, H. , Cai, D. , Ding, D. , & Xu, S. (2001). Primary studies on heroin abuse and injury induced by oxidation and lipoperoxidation. Chinese Medical Journal, 114(3), 297–302. https://www.ncbi.nlm.nih.gov/pubmed/11780318 11780318

[brb32397-bib-0080] Zisapel, N. (2001). Melatonin‐dopamine interactions: From basic neurochemistry to a clinical setting. Cellular and Molecular Neurobiology, 21(6), 605–616. 10.1023/A:1015187601628 12043836 PMC11533843

[brb32397-bib-0081] Zisapel, N. , Egozi, Y. , & Laudon, M. (1982). Inhibition of dopamine release by melatonin: Regional distribution in the rat brain. Brain Research, 246(1), 161–163. 10.1016/0006-8993(82)90157-3 7127086

[brb32397-bib-0082] Zisapel, N. , Egozi, Y. , & Laudon, M. (1983). Inhibition by melatonin of dopamine release from rat hypothalamus in vitro: Variations with sex and the estrous cycle. Neuroendocrinology, 37(1), 41–47. 10.1159/000123513 6684217

